# NON-INVASIVE PREVENTIVE VENTILATION WITH TWO PRESSURE LEVELS IN THE POSTOPERATIVE PERIOD OF ROUX-EN-Y GASTRIC BYPASS: RANDOMIZED TRIAL

**DOI:** 10.1590/0102-672020180001e1361

**Published:** 2018-06-21

**Authors:** Mabelle Gomes de Oliveira CAVALCANTI, Lívia Barboza ANDRADE, Patrícia Clara Pereira dos SANTOS, Leandro Ricardo Rodrigues LUCENA

**Affiliations:** 1 Institute of Integral Medicine Prof. Fernando Figueira, Recife, Brazil.

**Keywords:** Obesity, Bariatric surgery, Non-invasive ventilation, Postoperative complications, Obesidade, Cirurgia bariátrica, Ventilação não invasiva, Complicações pós-operatórias

## Abstract

**Background:**

Obesity is characterized by excessive accumulation of body fat, which causes damage to the health of individuals, such as breathing difficulties.

**Aim:**

To verify the results of non-invasive ventilation as a preventive strategy on the decline of respiratory function and postoperative complications in patients undergoing Roux-en-Y gastric bypass.

**Methods:**

This is a randomized trial, according to CONSORT standards, with obese adults aged 18-40 years. Randomized control group (n=25) only received guidelines regarding posture, early ambulation and cough stimuli, and in the NIV group (n=25), in addition to the aforementioned group, non-invasive ventilation was performed with two pressure levels, once day for 60 min, from the 1^st^ to the 3^rd^ postoperative day (POD). Both groups were evaluated in the preoperative period and in the 1^st^ and 3^rd^ POD for respiratory function, which were: slow vital capacity (VC), inspiratory capacity (IC), minute volume (MV), tidal volume maximal inspiratory muscle strength (Pimax) and peak expiratory flow (PEF). The length of hospital stay and the episodes of postoperative complications were recorded.

**Results:**

Of the 50 patients the majority were young adults with degrees of obesity between III and IV. In the intergroup analysis, there was an improvement in the CVL and MV only in the 1^st^ POD in the NIV group, CI in the three moments evaluated in the NIV group and the PFE in the 1^st^ and 3^rd^ PDO also in this group. The most frequent complications were pneumonia, followed by operative wound infection and atelectasis. There was a significant difference between groups, showing a higher occurrence in pneumonia and atelectasis in the control group. The days of hospitalization and intensive care unit were similar.

**Conclusion:**

It was observed a faster recovery until the 3^rd^ POD in the IC and PEF variables in the NIV group; in addition, there were fewer complications in this group.

## RESUMO

**Racional:**

A obesidade é caracterizada pelo acúmulo excessivo de gordura corporal, que acarreta prejuízos à saúde dos indivíduos, tais como dificuldades respiratórias.

**Objetivo:**

Verificar a efetividade da ventilação não invasiva, sobre o declínio da função respiratória e complicações pós-operatórias em pacientes submetidos ao bypass gástrico em Y-de-Roux.

**Métodos:**

Ensaio randomizado aberto, segundo padrões do CONSORT, com obesos, entre 18-40 anos. Foram randomizados em grupo controle (n=25) que receberam orientações quanto à postura, deambulação precoce e estímulo à tosse, e em grupo VNI (n=25) que além do citado, realizou ventilação não invasiva com dois níveis pressóricos, uma vez ao dia durante 60 min, do 1° ao 3º dia do pós-operatório (DPO). Ambos os grupos foram avaliados no pré-operatório e no 1^o^ e 3^o^ DPO quanto à função respiratória avaliando-se a capacidade vital lenta (CVL), capacidade inspiratória (CI), volume minuto (VM), volume corrente (VC), pressão inspiratória máxima (Pimáx) e pico de fluxo expiratório (PFE). O tempo de estadia hospitalar e os episódios de complicações pós-operatórias foram registrados.

**Resultados:**

Dos 50 pacientes avaliados na análise intergrupo, observou-se melhora da CVL e VM apenas no 1º ^)^DPO no grupo VNI, CI nos três momentos avaliados no grupo VNI e o PFE no 1º ^)^e 3º DPO também nesse grupo (p<0,05). As complicações mais frequentes foram pneumonia, infecção da ferida operatória e atelectasias; houve diferença significativa entre os grupos mostrando maior ocorrência na pneumonia e atelectasia no controle. Dias de internamento hospitalar, enfermaria e na unidade de terapia intensiva foram semelhantes.

**Conclusão:**

Houve recuperação mais rápida até o 3º DPO nas variáveis CI e PFE no grupo submetido à VNI, além de menos complicações pós-operatórias nesse grupo.

## INTRODUCTION

Obesity is characterized by excessive accumulation of body fat to such an extent that it impairs the health of individuals. However, the degree of excess fat, its body distribution and the health consequences vary among obese individuals^21^. According to the World Health Organization obesity is considered a public health problem, it is estimated that at least one billion people are overweight and approximately 300 million are obese, associating with a greater increase in indirect costs related to obesity. Absence from work, absenteeism and earlier retirements^23^.

Patients with BMI greater than or equal to 45 kg/m² present a decrease in life expectancy and an increase in cardiovascular mortality, which may reach 190%. In this context, bariatric surgery is a consistent resource in cases of severe obesity with documented failure of clinical treatment, providing patients with reduced mortality rates and improved quality of life^19^. In Brazil, the formal indications for gastric operations are: 18 to 65 years of age, BMI greater than 40 kg/m² or 35 kg/m² with one or more serious comorbidities related to obesity, and Roux-en-Y gastric bypass correspond to 75% of the procedures performed. According to a new balance sheet of the Brazilian Society of Bariatric and Metabolic Surgery, in 2015 93.5 thousand people were submitted to the procedure, an increase of 6.25% in relation to the previous year^23^.

Abdominal surgical procedures, especially postoperative gastroplasty, affect the respiratory muscles through different mechanisms, such as loss of muscle integrity through surgical incision, use of neuromuscular blockers during anesthesia, and pain, favoring a decrease in volumes and capacities pulmonary diseases. This fact leads to inspiratory overload, which leads to lower muscle strength and endurance. Additionally, the accumulation of adipose tissue in the abdomen and rib cage gives biomechanical disadvantage to the diaphragm^18^.

The incidence of clinically relevant pulmonary complications in the postoperative period of abdominal operations ranges from 5-30% ^6^ ^,^ ^7^. These are the main causes of morbidity and mortality, increasing hospitalization time, medication use and hospital cost ^22^ ^,^ ^27^.

The use of non-invasive ventilation (NIV) is currently method capable of offering positive pressure, which is useful in increasing oxygenation, reducing respiratory complications, and increasing the incidence of anastomosis dehiscences^1^. NIV can be offered in the two-level (bilevel) or positive airway (CPAP) mode and is an alternative for the prevention of pulmonary complications, as it decreases muscle fatigue, improves functional residual capacity (FRC), reduces areas of intrapulmonary shunt through the recruitment of collapsed alveolar units, aiming at the adequate maintenance of gas exchange, facilitating alveolar ventilation and reducing dyspnea, reducing respiratory work ^1^ ^,^ ^2^ .

The objective of this study was to verify the effectiveness of NIV as a preventive strategy on the decline of respiratory function and postoperative complications in patients undergoing Roux-en-Y gastric bypass.

## METHODS

This is a randomized clinical trial in patients with degrees III and IV obesity, aged 18-40 years, submitted to gastroplasty performed at the Institute of Integral Medicine Prof. Fernando Figueira (IMIP) in Recife, PE, Brazil, from October 2013 to March 2015. Patients signed an informed consent form after the guidelines on the proposed protocol, which was approved by the Research Ethics Committee of the institution under number 4064 -14. Patients with hemodynamic instability, presence of contraindication to the use of NIV, chronic lung disease or unfitness for the evaluation techniques were excluded.

In the preoperative period, the following characteristics were evaluated: age, gender, height, weight, body mass index and respiratory function, tidal volume (VT), respiratory rate (RP), minute volume, inspiratory capacity (IC), peak expiratory flow (PEF) and maximal inspiratory pressure (MIP). Regarding the surgical procedure, data such as anesthesia time, mechanical ventilatory assistance (MVA) and postoperative complications were obtained.

After the surgical procedure, the patients were extubated within 24 h and randomized by computerized program into two groups: G1, control (n=25) and G2, NIV (n=25). The allocation was performed randomly and hidden in sealed opaque envelopes containing the name of each group.

The G1 group received guidelines regarding posture, early ambulation and cough stimulation. It was recommended to avoid antalgic positions (increased thoracic syphosis, shoulder protrusion, and flexion of the head) due to surgical incision, since they could compromise respiratory function. Early ambulation was encouraged and patients were instructed to cough by securing the surgical incision with their hands supported on it, providing greater safety and greater cough efficacy.

The G2 group, in addition to the aforementioned guidelines, underwent non-invasive ventilation with two airway pressure levels (bilevel) once daily for 60 min from the 1^st^ to 3^rd^ postoperative day, with Respironics^® )^portable NIV device (Bipap Synchrony II), coupled to the nasal mask. The parameters were adjusted, aiming at target tidal volume of 7 ml/kg of predicted weight, limiting the inflation pressures in 20 cm H_2_O, with IPAP ranging from 14-16 cm H_2_O and fixed EPAP 7 cm H_2_O.

Respiratory function variables were reevaluated in the 1^st^ and 3^rd^ postoperative days, and the hospital stay and ICU were later obtained. Pulmonary function tests were evaluated with the patients sitting in the bed. Ventilatory variables were measured using an analog ventilator (nSpire health Inc^®)^, Longmont, USA) coupled with a face mask. PEF was measured through the portable peakflow (Clement Clark^®)^, England, mini-wright model), coupled to a mouthpiece and using a nasal clip in the patient, after maximal inspiration and forced exhalation with open glottis. In order to measure MIP, an analogical manovacuometer (Comercial médica^®)^) was used, coupled to the mouthpiece and using a nasal clip in the patient, through a maximum inspiration from the CRF. To guarantee reliability of the measurement, three attempts were made, among which the highest value was recorded.

### Statistical analysis

Was performed using software SPSS 19 and R-project. The data were exposed in means and standard deviation and error. The comparison of means of numerical variables was performed with Student’s t-test. To verify the possible differences between the frequencies, the chi-square test (chi-square test) and the variance analysis test (Anova) were used to verify differences between means in relation to the evaluated periods. A significance level of 0.05 was adopted.

## RESULTS

**FIGURE 1:**
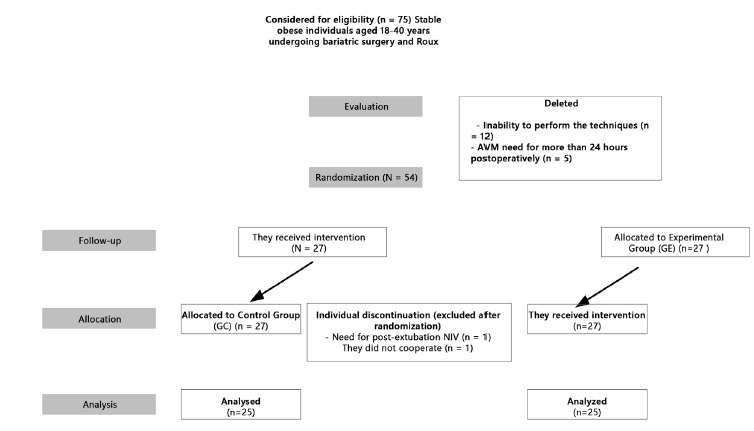
Flowchart for monitoring and capturing participants (CONSORT^25^)

Of the 75 patients eligible for bariatric surgery 54 were randomized and 50 completed the study as presented in the follow-up and follow-up flowchart of the participants (Figure 1).

The baseline data for the sample are shown in Table 1.

**TABLE 1:** Baseline characteristics of the sample

Variables	All patients (n=50)	Control group (n=25)	Experimental group (n=25)	p
Age	29.67±7.27	28.68±8.11	30.62±6.38	0.418
BMI	48.34±6.24	49.26±6.87	47.46±5.56	0.349
Anesthesia Time(min)	282.55±36.87	279.6±39.21	285.38±35.01	0.271
AVM time (min)	260.98±36.95	258.8±38.11	263.08±36.42	0.198
ICU day (d)	1.45±0.5	1.48±0.51	1.42±0.50	0.528
Days of hospitalization(d)	3.18±0.48	3.24±0.44	3.12±0.52	0.459
Hospital stay time(d)	4.65±0.84	4.72±0.84	4.58±0.86	0.677

Chi-square test. p<0.05 *.

Regarding the general anthropometric characteristics in the preoperative period, the mean age of the patients was 29.67±7.27 years, the majority of young adults composed of 58.8% men, the mean body mass index was 48.34±6.24, degree of obesity III and IV, with no significant difference between groups.

The mean number of days of anesthesia and mechanical ventilation, number of days in the ICU, ward and hospital stay are shown in Table 1. There was no difference between the groups.

Table 2 shows the comparisons between the control and NIV groups in the three moments of the evaluation - preoperative, 1^st^ and 3^rd^ postoperative day (POD) - of pulmonary function variables and muscle strength. It was observed in this study a higher CVL in the 1^st^ POD, the higher CI in the pre-, 1^st^ and 3^rd^ POD moments, the highest MV in the 1^st^ POD and the highest PEF in the 1^st^ and 3^rd^ POD in the group submitted to the NIV. 

**TABLE 2:** Comparison between control and NIV groups in relation to lung function and inspiratory muscle strength

Variables	Control group (n=25)	NIV group (n=25)	p
Inspiratory pressure			
Preoperative	110,4±11,72	106,69±13,95	0,310
1POD	93,48±19,49	99,42±18,67	0,271
3POD	103,24±14,62	108,65±13,97	0,182
Respiratory frequency			
Preoperative	16,36±2,40	16,88±2,61	0,459
1POD	19,24±2,82	18,81±4,40	0,677
3POD	15,56±1,61	15,96±1,87	0,415
Slow vital capacity			
Preoperative	1842,69±505,42	1782,6±498,35	0,671
1POD	1033,85±253,16	1366,4±380,79	0,001*
3POD	1384,23±391,65	1541,8±415,68	0,170
Inspiratory capacity			
Preoperative	1825,38±418,25	2197,6±490,01	0,005*
1POD	1125,0±208,0	1474,4±327,63	<0,0001*
3POD	1334,23±270,26	1557,6±266,94	0,005*
Minute volume			
Preoperative	9,27±1,57	9,44±1,31	0,666
1POD	9,22±1,20	9,97±1,33	0,039*
3POD	9,37±1,21	9,61±1,01	0,450
Tidal volume			
Preoperative	590,16±136,37	572,08±111,31	0,606
1POD	487,84±98,86	560,77±154,66	0,050
3POD	612,32±125,07	610,85±92,18	0,962
Peak expiratory flow			
Preoperative	219,62±32,74	238,6±46,84	0,102
1POD	112,88±23,29	132,4±36,94	0,030*
3POD	148,65±32,84	203,92±53,76	<0,0001*

POD=postoperative day; NIV noninvasive ventilation; * =Student’s t-test p <0.05

The main postoperative complications observed by the patients were pneumonia, the most common event affecting 21.2% of the sample, followed by operative wound infection 13.5% and atelectasis 9.6%. The proportion of patients presenting two or more complications corresponded to 11.5% and without complications 42.3%. It was observed that in the control group there were more events of pneumonia and atelectasis (p=0.001 and p=0.005, respectively, Table 3).

**TABLE 3:** Frequency distribution of postoperative complications in relation to the analyzed groups

Complications	Control n%	NIV n%	p
Pneumonia	8 - 58,5	5 - 45,5	0,001*
Surgical wound infection	4 - 57,1	3 - 42,9	0,210
Atelectasis	4 - 80,0	1 - 20,0	0,005*
Anastomosis ulcer	1 - 100,0	0 - 0,0	0,182
Two or more complications	3 - 50,0	3 - 50,0	0,968
None	7 - 31,8	1568,2	0, 030*

NIV=noninvasive ventilation; * =Chi-square test. P <0.05

Comparison of total hospital, nursing and ICU stay times did not show a significant difference between the studied groups (Table 4).

**TABLE 4:** Comparison of hospitalization time, ward and ICU between Control and Non-Invasive Ventilation (NIV) groups

Variables	Control group (n=25)	NIV group (n=25)	p
ICU (days)	35.52±12.23	34.15±12.09	0.690
Ward (days)	77.76±10.46	74.77±12.38	0.357
Hospitalization	113.28±20.22	109.85±20.56	0.551

NIV=noninvasive ventilation; ICU=intensive care unit; * =Student’s t-test. p <0.05.

## DISCUSSION

In the studied sample, a slow and inspiratory vital capacity increase was observed in the group submitted to NIV in a preventive way in the postoperative period, in addition to an improvement in peak expiratory flow with no impact during hospital stay times.

Continuously or intermittently administered NIV has been used alone or in combination with other therapies to prevent atelectasis and hypoxemia and to increase lung capacity during the postoperative period of gastroplasty^13^. To date, this study represents the first randomized trial to evaluate NIV in the bilevel mode on a preventive basis on respiratory function and postoperative complications in adults undergoing Roux-en-Y gastric bypass.

Regarding the characteristics of the sample, the participants were mostly young, with a slight predominance of males, with a mean age of 30 years, with degree of obesity III, IV and mean length of hospital stay of four days. These data are different from a retrospective descriptive study by Ramos^24^, which showed that obesity was more prevalent in women with a mean age of 43 years with mild to moderate obesity and total hospital stay in three days.

In the present study, pulmonary function (inspiratory capacity and peak expiratory flow) returned more rapidly to baseline on the third postoperative day in the NIV group. The comparison of the variables of intergroup respiratory function over time in this study confirms the results found by Baltieri ^1^. They performed a randomized trial with 44 BMI patients between 40-55 kg/m^2^ and mean age 40 years, who, after being submitted to Roux-en-Y gastric bypass, had increased lung capacity in the group receiving the NIV after the 2^nd^ POD, due to less loss of expiratory reserve volume, and increased thoracic incursions and cough efficacy.

However, inspiratory muscle pressure (Pimax) did not return to preoperative values ​​in the 3^rd^ postoperative day, in both groups, perhaps because it is too short a time for surgical recovery. In the study by Pelozzi^19^, the muscle strength analyzed only returned to baseline values ​​after six weeks of surgical intervention, demonstrating the need for a longer recovery time, considering that respiratory muscle strength increases directly with the patient’s clinical improvement in the postoperative period. It is likely to be related to the decrease of pain and the improvement of the elastic components of the rib cage resulting from the cicatrization process^16^.

Regarding MV, a significant difference was observed in the 1^st^ POD in the NIV group although this difference was not sustained on the 3^rd^ day; this can be explained by the fact that the MV is the result of a product of the VT by the RP and such variables showed a significant decrease and increase, respectively, during the evaluations, the anesthesia promotes an increase in the alveolar-arterial gradient difference, which should be compensated with the increase of ventilation in an attempt to maintain adequate arterial oxygenation^17^. This fact was also observed in a similar study, performed with 36 patients of both genders, using CPAP as the mode of NIV up to 48 h post-extubation of the postoperative period of bariatric surgery, with no significant statistical difference of the MV in the first days postoperative compared to preoperative values^18^.

In the comparison between groups, PEF showed a significant difference in the NIV group during the two postoperative periods analyzed. The PEF variable is related to the degree of airway obstruction and cough efficacy, the greater the latter, the better the elimination of secretions and, consequently, fewer postoperative pulmonary complications^19^. This finding corroborates the study by Ebeo ^6^, and can be explained by the increase in functional residual capacity (FRC) provided by the use of positive pressure, thus generating a higher pulmonary volume and a consequent increase in expiratory flow^20^.

However, on the respiratory function, the randomized study of Forgiarini^8^ e Remístico^25^ evaluated lung volumes and capacities in obese patients of both genders, mean age 35 years, and used non ventilation invasive surgery in the immediate postoperative period of gastroplasty, demonstrating a significant difference in pulmonary function variables between the groups.

This fact corroborates these results, due to the fact that the slow and inspiratory vital capacity has a reduction of up to 30% in the obese degrees III and IV, generating increased respiratory work due to mechanical diaphragmatic disadvantage and loss of the elastic component of the thoracic cavity. Therefore, the NIV with two pressure levels in this population profile, applied in the first 24 h postoperatively, significantly reduce restrictive changes^23^.

The complications observed in the postoperative period in this analysis corresponded to 38%, being above the study by Futie^30^, in which 35% of patients had some type of complication after bariatric surgery. The frequency distribution of respiratory complications, such as pneumonia and atelectasis when compared to the control group, was lower in the intervention group, this data corroborates Tenorio^28^ in which the experimental group experienced fewer complications. This fact could be justified due to the restoration of functional residual capacity, preventing collapse of the lower airways and increasing pulmonary complacency^25^. 

Vassilakopoulos analyzed the respiratory complications after laparotomic bariatric surgery and showed that pneumonia and atelectasis are the most prevalent, being in agreement with these results. They may be caused by decreased mucociliary clearance, reduced bed mobility, decreased secretion, associated with changes in the physiological, diaphragmatic respiratory pattern, for more superficial and predominantly thoracic breathing, culminating in a lower efficacy of cough and accumulation of pulmonary secretions^29^. The use of NIV promotes an increase in the compliance of the respiratory system by reversing pulmonary microatelectasis, reducing respiratory work with effectiveness in reducing pulmonary complications and increasing gas exchange^27^ ^,^ ^28^. In this analysis, no significant differences were observed in hospital, ward and ICU stay between groups. Recently Lieschinget & Chen in a multicenter clinical study, analyzed 86 patients undergoing bariatric surgery and use of NIV in the postoperative period and did not show a significant reduction in mortality or hospital length of stay^3^ ^,^ ^14^.

Relevant limitations of this study are: the short follow-up time of patients, changes in the standardization of hospital discharge, shortening patients’ stay time and the fact that there is no blindness of the evaluator in relation to the groups evaluated.

Future investigations, with new randomized trials using different NIV protocols, with longer duration and/or frequency, may show increased volumes, lung capacities, being able to demonstrate greater gains in respiratory function and in the length of hospital stay of these patients.

## CONCLUSION

Patients in the postoperative period of gastric bypass in Roux-en-Y had a faster recovery in inspiratory capacity and peak expiratory flow in the group submitted to preventive NIV. In addition, there were fewer postoperative complications in this group. No difference was observed in the time of hospitalization and intensive care.
